# Associations Between the Maternal Diet Index and Childhood Asthma: The NorthPop and Healthy Start Cohorts

**DOI:** 10.1002/clt2.70144

**Published:** 2025-12-18

**Authors:** Stina Bodén, Carina Venter, Kaci Pickett‐Nairne, Deborah H. Glueck, Richard Lundberg‐Ulfsdotter, Magnus Domellöf, Dana Dabelea, Christina E. West

**Affiliations:** ^1^ Department of Clinical Sciences, Pediatrics Umeå University Umeå Sweden; ^2^ Section of Allergy & Immunology, Department of Pediatrics University of Colorado Denver School of Medicine Aurora Colorado USA; ^3^ Children’s Hospital Colorado Aurora Colorado USA; ^4^ Department of Pediatrics University of Colorado School of Medicine University of Colorado Denver Aurora Colorado USA; ^5^ Lifecourse Epidemiology of Adiposity and Diabetes Center University of Colorado Anschutz Medical Campus University of Colorado Denver Aurora Colorado USA; ^6^ Department of Epidemiology, Colorado School of Public Health University of Colorado Denver Aurora Colorado USA

**Keywords:** asthma prevention, childhood allergic disease, maternal diet, MDI, nutrition

## Abstract

**Background:**

A novel maternal diet index (MDI), characterizing offspring asthma‐ and allergy‐associated maternal intake during pregnancy was constructed and validated in Healthy Start, USA. This study aims to (1) externally validate the asthma findings from Healthy Start in the NorthPop Birth Cohort Study (NorthPop) in Sweden; and (2) characterize the diet and demographics of the two cohorts.

**Methods:**

The MDI was computed as a weighted combination of seven components associated with offspring allergies and asthma, including vegetables and yogurt (associated with decreased odds) and cold cereals, fried potatoes, juice, red meat, and rice (associated with increased odds). Doctor diagnoses provided childhood asthma incidence and timing. Parametric Weibull time‐to‐event analysis evaluated associations between the MDI, dichotomized at the median (72.2) for Healthy Start, and offspring asthma.

**Results:**

The NorthPop and Healthy Start mean MDI values differed significantly (*p* < 0.001) and in NorthPop, only 6.1% had MDI < 72.2. Data from 6446 mother‐child dyads in NorthPop yielded a crude hazard ratio (HR) for asthma of 0.70 (95% confidence interval [CI] 0.50–0.98, *p* = 0.037) and a fully adjusted HR of 0.84 (0.55–1.29; *p* = 0.428) for MDI > 72.2 versus < 72.2 (*n* = 4655). The fully adjusted HR for 945 Healthy Start dyads was significant at HR 0.41 (0.29–0.57; *p* < 0.0001).

**Conclusions:**

Results show that in a population with different maternal dietary patterns and demographics compared to the source population, MDI > 72.2 was not an independent predictor of offspring asthma. Further proof of the utility and generalizability of the MDI needs to be tested in other populations.

## 1 Background

The rising prevalence of childhood asthma, particularly in high‐incidence regions such as the United States of America (USA) [1] and Northern Europe, including Sweden [2] places a high burden on affected children and their families. Although significant progress has been made in elucidating the pathophysiology of asthma, effective strategies for both primary and secondary prevention remain insufficient [3]. Given the substantial impact of asthma on patients’ quality of life and its associated economic burden [4], the development and implementation of preventive measures are of great importance.

Since lung development begins in pregnancy, the Global Initiative for Asthma (GINA) suggests preventing asthma “*in utero*” [3]. GINA reviewed primary prevention strategies for asthma, including maternal nutrition. They concluded that the evidence for previously studied strategies, including dietary changes in pregnancy designed to prevent offspring asthma, is limited.

A meta‐analysis of Venter et al. showed limited or no associations between maternal dietary patterns or intake of single nutrients or foods during pregnancy and primary prevention of asthma and recurrent wheeze [5]. One possible reason for the lack of findings in dietary prevention [6, 7, 8, 9, 10] was that previous studies on dietary prevention approaches focused on dietary indices initially developed in adult populations to study associations with cardiometabolic diseases and cancer [11, 12, 13].

By contrast, the maternal diet index (MDI) was designed specifically to be associated with reduced risk of offspring allergic diseases [14]. Venter et al. developed the diet index using a test‐and‐replication approach [14, 15, 16, 17, 18], using data on maternal diet and offspring allergic diseases and asthma from Healthy Start, a pre‐birth cohort in Colorado, USA (*N* = 1410 mother‐offspring dyads) [14]. The diet associated with reduced risk of offspring allergies and asthma is high in intake of vegetables and yogurt (two components associated with reduced risk), and has reduced intake of cold cereals, fried potatoes, fruit juice, red meat, and rice (five components associated with increased risk).

While Venter et al. showed associations between the MDI and risk of asthma [14], the index has not yet been validated in other cohorts. To externally validate the proposed association, it is important to apply the MDI in additional populations with a high prevalence of childhood asthma. Therefore, the aims of this study were to (1) externally validate the MDI by investigating associations to childhood asthma in Healthy Start and in the NorthPop Birth Cohort Study (NorthPop) and (2) characterize the diet and demographics in the two cohorts.

## 2 Methods

### 2.1 Cohorts

#### 2.1.1 Healthy Start

Healthy Start is a longitudinal observational study [19] registered at ClinicalTrials.gov (NCT02273297). Mothers were recruited from prenatal clinics at the University of Colorado Hospital, in Denver Colorado, between 2009 and 2014. Written informed consent was obtained before the first research visit. The Colorado Multiple Institutional Review Board approved the study. Mothers were excluded if they had severe and life‐threatening illnesses, including asthma treated with steroid medications, cancer, pre‐existing diabetes, or diagnosis of psychiatric illness, were pregnant with multiple offspring, had a previous stillbirth, were younger than 16 years of age during the consent process, or had gestational age at the baseline visit of more than 24 weeks [19]. Participant data were excluded if the participant withdrew consent, had a fetal death, or gave birth at less than 25 weeks of gestational age.

#### 2.1.2 NorthPop

NorthPop is a large ongoing longitudinal observational prospective, population‐based birth cohort study with recruitment in pregnancy [20]. Ethical permission was granted by the regional Ethics Committee in Umeå, Sweden 2014/224–31 with amendments (2016/349‐31 and 2018/504‐32) from the Ethical Review Authority. Informed consent was collected verbally, and in written format from both parents. NorthPop invites all pregnant women and partners meeting eligibility conditions in the Umeå University Hospital catchment area, including three Study sites (Umeå, Skellefteå, and Lycksele) in Västerbotten County. For the current study, data were obtained from women recruited to NorthPop between May 2016 and December 2023. Mothers were eligible if they were ≥ 18 years of age, had a viable pregnancy, were able to understand the Swedish language, and intended to give birth and reside in the catchment area for the next few years. At the time of recruitment, the pregnancy had to be between 14 and 24 weeks of gestation as determined by ultrasound examination. The analytic sample in this study included pregnant mothers with completed food frequency questionnaires (FFQ) and children from singleton pregnancies born by December 2023.

### 2.2 Maternal Diet

#### 2.2.1 Healthy Start

Maternal diet was assessed during mid‐pregnancy and at delivery. A 41 item Food Propensity Questionnaire (FPQ) inquired about intake of both food and drink items. The questionnaire is published in full detail in the Supporting Information S1 of Venter et al. [14].

The macro nutrients were estimated using the mean values from the Nutrition Data System for Research (NDSR) reports of repeated automated self‐administered 24‐h dietary recalls (ASA24) and summarized as amount per day.

#### 2.2.2 NorthPop

Maternal dietary intake and portion‐size were collected around gestational week 35 using a web‐based, 125‐item FFQ [21, 22]. Solid foods were reported in five frequencies: never or rarely, 1–3 times per week, 4–6 times week, 1–2 times/day, or ≥ 3 times/day. Separately, bread type and frequency of consumption was reported, ranking bread types from 1 (most often consumed) to 6 (least often consumed). Beverages were reported in seven frequencies: never or rarely, 1–3 glasses per month, 1–3 glasses per week, 4–6 glasses per week, 1–2 glasses per day, 3–4 glasses per day, and ≥ 5 glasses/day. Cow's milk and milk substitute totals were reported in deciliters per day. Maternal reported intake frequencies, maternal reported usual portion size of carbohydrate‐dense foods, protein‐dense foods and vegetables, as well as standard portion sizes [23] were converted into daily intakes in quantity per day using the Swedish Food Agency's food database [24].

### 2.3 Maternal Diet Index

#### 2.3.1 Healthy Start

The maternal diet index is a sum of scaled intake of vegetables, yogurt, fries/fried potatoes, rice/grains, red meats, 100% fruit juices, and cold cereals. The computation was conducted using methods and code described by Venter et al. [14]. The MDI components were estimated from the maximum of two FPQs and summarized as daily intakes [14].

#### 2.3.2 NorthPop

Variables from the FFQ were chosen and aggregated to match the seven components of the MDI. As for Healthy Start, the MDI was computed using methods and code described by Venter et al. [14].

### 2.4 Childhood Asthma Ascertainment

#### 2.4.1 Healthy Start

As described in Venter et al. [25], incidence and timing of asthma were assessed using an electronic medical record search strategy and including at least one hit for asthma diagnosis. The search leveraged multiple independent reviews, with consensus conference used to resolve unclear diagnoses.

#### 2.4.2 NorthPop

Incidence and timing of physician‐diagnosed asthma was ascertained including participants with at least one ICD‐10 code J45x obtained from the Swedish National Patient Register [26] reported from the birth of the child until November 2024.

### 2.5 Variables Used to Characterize the Cohorts, and Covariates Used in the Models

#### 2.5.1 Healthy Start

Pre‐pregnancy maternal body mass index (BMI) was obtained from electronic medical records (81.5%) or self‐reported (12.5%) [27]. Maternal age, maternal university education (yes/no) and gestational age at birth were obtained from the baseline and post‐natal questionnaires.

Model covariates were chosen from a large set of covariates from the literature, described by Venter et al. [14]. Maternal questionnaires collected early in pregnancy provided data on sociodemographic history of maternal asthma and parity (as first child vs. later child) [16]. Questionnaires at approximately 27 weeks of gestation provided self‐reported information on tobacco smoking during pregnancy. Offspring electronic medical records described mode of delivery and child's sex. A questionnaire at the 18‐month post‐natal interview recorded the presence of breastfeeding at age 6 months and whether solid foods were introduced before 4 months of age.

#### 2.5.2 NorthPop

Web‐based questionnaires completed at gestational age 14–24 and 26 weeks provided information on maternal tobacco smoking in pregnancy, maternal university education, and maternal history of asthma. The Swedish Pregnancy Register [22] provided data on parity, maternal BMI measured at the first visit to the maternity center, and mode of delivery. Maternal age at delivery, gestational age, and offspring sex was extracted from the NorthPop database. If the child was breastfed at age 6 months (both exclusively and partly) as well as information about the timing of complementary food introduction were both obtained from parental questionnaires completed when the child was around 9 months of age.

### 2.6 Statistical Analysis

#### 2.6.1 Software

Analyses were conducted using IBM SPSS Statistics, version 29.0 (IBM Corp, 2021, Armonk NY), SAS version 9.4 (SAS Institute Inc., 2013), and R version 4.4.2 (R Foundation for Statistical Computing, Vienna, Austria). A *p* value < 0.05 was considered statistically significant.

#### 2.6.2 Demographic Data

Demographic data were reported for each cohort, and within each cohort, dichotomized by median MDI in Healthy Start [14]. *C*ontinuous data, including dietary intake data, was reported as mean (± SD) and compared between cohorts using Satterthwaite t‐tests. Categorical data are presented as frequencies and proportions and compared between cohorts using Fisher exact tests.

#### 2.6.3 Associations Between Maternal Diet and Risk of Offspring Childhood Asthma

For each cohort parallelly, we fit a planned sequence of unadjusted, partially adjusted, and fully adjusted Weibull time‐to‐event models with the time to age at first diagnosis of asthma as the outcome, and MDI dichotomized by the median value for Healthy Start as the predictor, as previously described [16, 17]. Women with MDI scores greater than the Healthy Start median were classified as having allergy and asthma preventive diets [16]. This cutoff was used to preserve comparability across studies and facilitate direct interpretation in relation to previous [16, 17] and future studies. We provided model‐based unadjusted and adjusted hazard ratios (HR), and 95% confidence intervals. Participants with no reported asthma were considered to be right censored at the end of follow‐up, which differed depending on parental permission in Healthy Start, and the date of birth in NorthPop. Median and interquartile range (IQR) of follow‐up time were computed for both cohorts. The partially adjusted model was controlled for maternal asthma while fully adjusted model included the covariates maternal asthma, gestational smoking, gestational energy intake, if first born child, mode of delivery, child sex, presence of breastfeeding at age 6 months, and if early introduction of any solid foods (< 4 months). We used predicted cumulative distribution (CDF) curves to illustrate the average of predicted asthma proportions over time for each dichotomized group, estimated with categorical covariates at their most frequently occurring value and continuous covariates at their mean level.

## 3 Results

### 3.1 Analytic Sample

**FIGURE 1:**
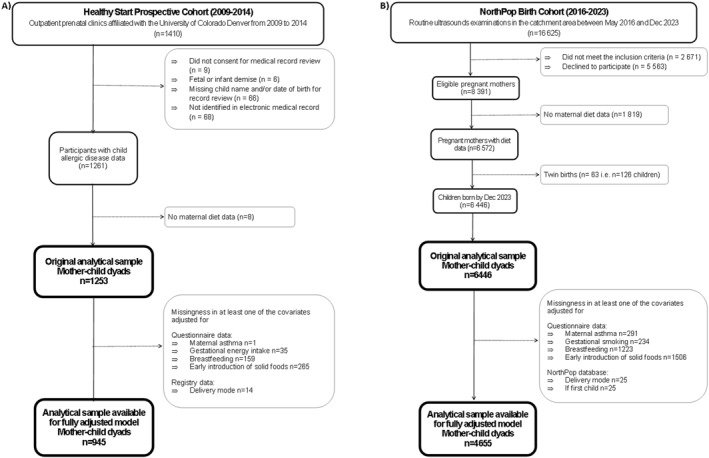
Participant diagrams for Healthy Start (A), and NorthPop (B).

The crude analytic sample for NorthPop and Healthy Start included 6446 and 1253 mother‐child dyads, respectively, and the final analytical sample included 4655 and 945 mother‐child dyads, respectively (Figure 1).

### 3.2 Demographic Comparison

The two cohorts differed significantly on a number of characteristics (Table 1 and stratified by median MDI in Supporting Information S1: Tables S1 and S2). The NorthPop population had a higher gestational age (*p* < 0.0001), lower maternal mean BMI (*p* = 0.007) and higher maternal age (*p* < 0.0001) compared to the Healthy Start population (Table 1). In NorthPop, a smaller proportion of the pregnant women smoked (*p* < 0.0001) and had a caesarean section (*p* < 0.0001). Breastfeeding (partly or exclusive) at age 6 months was more prevalent in NorthPop (82.7%) than Healthy Start (61.4%) (*p* < 0.0001). A smaller proportion of infants in NorthPop were introduced to solid foods before 4 months of age compared to the Healthy Start population (*p* = 0.04), (Table 1). There were no statistical differences between the two study populations with regard to child sex, percentage of first‐born children, and history of maternal asthma (Table 1).

**TABLE 1:** Characteristics of Healthy Start and NorthPop participants.

	Healthy Start	NorthPop	*p* value^b^
	All participants *N* = 1253^a^	All participants *N* = 6446^a^	
Categorical variables, *n* (%)			
Any university education	754 (60.2)	4092 (66.3)	< 0.0001
Maternal history of asthma	208 (16.6)	1132 (18.4)	0.14
Smoking in pregnancy	106 (8.5)	75 (1.2)	< 0.0001
First born child	594 (47.4)	3017 (47.0)	0.80
Caesarean section	270 (21.8)	1062 (16.5)	< 0.0001
Female sex child	600 (47.9)	3172 (49.2)	0.40
Breastfeeding at age 6 months	672 (61.4)	4319 (82.7)	< 0.0001
Introduction of solid food by age 4 months	149 (15.1)	490 (9.9)	0.04
Continuous variables, mean (SD)			
Maternal pre‐/early pregnancy BMI (kg/m^2^)	25.7 (6.2)	25.2 (4.8)	0.007
Maternal age at delivery (years)	27.8 (6.2)	31.0 (4.4)	< 0.0001
Gestational age at birth (weeks)	39.3 (1.7)	39.5 (1.4)	< 0.0001

Abbreviations: BMI, body mass index; SD, standard deviation.

^a^ Characteristics by the Healthy Start MDI median and missingness for both study populations are presented in Supporting Information S1: Tables S3 and S4.

^b^ Fisher exact test for categorical variables and Satterthwaite *t*‐test for continuous variables.

### 3.3 Dietary Intake

**FIGURE 2:**
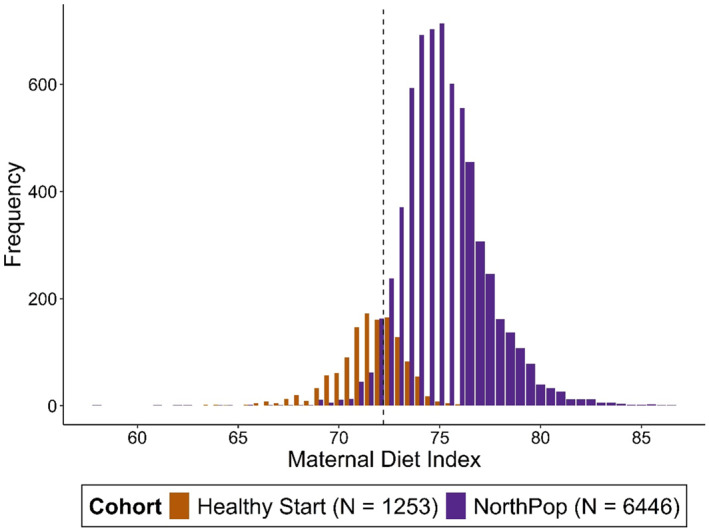
Distribution of maternal diet index (MDI) scores in Healthy Start (*n* = 1253) and NorthPop (*n* = 6446), respectively, displayed with histograms. A vertical line at 72.2 represents the cutoff for “low” and “high” dichotomizations of the MDI values.

Compared to pregnant women in Healthy Start, pregnant women in NorthPop reported lower intakes of cold cereals, fried potatoes, juice, and rice, and higher intakes of red meat, vegetables, and yogurt (Table 2). Women in NorthPop had higher average daily reported energy, carbohydrate, protein, and fiber intake, and lower average fat intake per day (Table 2). Only 6.1% (*n* = 391) of the pregnant NorthPop population had MDI scores less than the Healthy Start median MDI score of 72.2 (Supporting Information S1: Table S2). In Figure 2, the distributions show a lower distribution of scores in Healthy Start compared to Northpop, with overlapping histograms, also demonstrating that MDI scores were normally distributed within the two populations.

**TABLE 2:** Maternal dietary intake in pregnancy presenting the seven foods included in the maternal diet index (MDI), the total MDI, and macronutrient intake in Healthy start and NorthPop.

	Healthy Start *N* = 1253	NorthPop *N* = 6446	
Foods included in MDI	Mean (SD) daily intake, frequency	Mean (SD) daily intake, frequency	*p* value^a^
Cold cereal	0.69 (0.52)	0.17 (0.38)	< 0.0001
Fried potatoes	0.27 (0.32)	0.15 (0.18)	< 0.0001
Juice	0.94 (1.21)	0.42 (0.60)	< 0.0001
Red meat	0.40 (0.38)	0.81 (0.69)	< 0.0001
Rice	0.40 (0.38)	0.37 (0.32)	0.009
Vegetables	0.91 (0.65)	3.29 (1.86)	< 0.0001
Yogurt	0.53 (0.46)	0.81 (0.80)	< 0.0001
MDI, total	72.1 (1.69)	74.91 (2.09)	< 0.0001

**Macronutrients**	**Mean (SD) daily intake, quantity (*N* = 1221)**	**Mean (SD) daily intake, quantity (*N* = 6446)**	
Energy intake, kcal/day	2062.12 (384.28)	2194.40 (788.54)	< 0.0001
Carbohydrates, g/day	256.16 (94.21)	293.86 (99.53)	< 0.0001
Protein, g/day	82.50 (29.94)	101.39 (43.69)	< 0.0001
Fat, g/day	80.35 (34.32)	74.45 (30.49)	< 0.0001
Fiber, g/day	18.32 (8.48)	27.11 (14.76)	< 0.0001

^a^ Satterthwaite *t*‐test.

### 3.4 Associations Between Maternal Diet Index, and Asthma Outcomes

**FIGURE 3:**
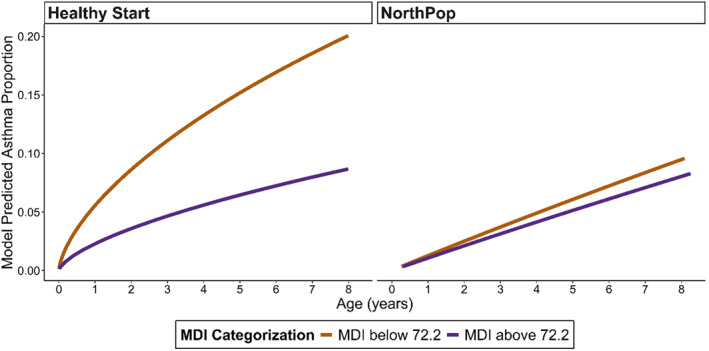
Parametric fully adjusted Weibull time‐to‐event models for associations between dichotomized maternal diet index (MDI) (below/above 72.2) and offspring asthma outcomes for Healthy Start (left) including *n* = 945 dyads and NorthPop (right) including *n* = 4655 dyads. The predicted cumulative distribution (CDF) curve illustrates the average of predicted asthma proportions over time after adjusting for history of maternal asthma, gestational smoking, gestational energy intake, caesarean section, first born child, child sex, any breastfeeding at age 6 months, and introduction of solid foods by 4 months. Median follow‐up after full adjustments for Healthy Start was 5.2 years (4.0, 6.4), and for NorthPop 4.3 years (2.7, 5.8).

Median follow‐up for 1253 Healthy Start participants was 4.9 years (4.0, 6.5; Supporting Information S1: Table S5), and for 6446 NorthPop participants 4.2 years (2.7, 5.8; Supporting Information S1: Table S6). Both in NorthPop and in Healthy Start, higher MDI scores dichotomized by the median from Healthy Start, were associated with significantly lower hazards of offspring asthma, in unadjusted models (HR 0.70; 95% CI 0.50–0.98. *p* = 0.037 and HR 0.36; 95% CI 0.27–0.49. < 0.0001, respectively; Table 3). After multivariable adjustments, estimates remained significant in Healthy Start (HR 0.41; 95% CI 0.29–0.57; *p* < 0.0001; Table 3) and the adjusted parametric Weibull time‐to‐event model demonstrated a difference in asthma incidence between children where the pregnant woman had an MDI score below the median (72.2) compared to children whose mothers had an MDI above the median (Figure 3). In the NorthPop population, this was not the case (Figure 3), and hazards were non‐significantly different in the fully adjusted models (HR 0.84; 95% CI 0.55–1.29; *p* = 0.428) (Table 3). Sensitivity analysis for asthma ascertainment in the NorthPop population restricted to at least two physician‐diagnosed asthma ICD‐10 codes (*n* = 319), yielded stronger associations between MDI and offspring asthma in all three models (Supporting Information S1: Table S7), but as before, significant HRs only in the unadjusted and partially adjusted models. Using the NorthPop specific median for dichotomization of the MDI showed no associations to offspring asthma (Supporting Information S1: Table S8).

**TABLE 3:** Dichotomized maternal diet index (MDI) in pregnancy and unadjusted and adjusted associations with hazard of childhood asthma diagnosis. The partially adjusted model adjusts for history of maternal asthma. The fully adjusted model additionally adjusts for gestational smoking, gestational energy intake, caesarean section, first born child, child sex, any breastfeeding at age 6 months, and introduction of solid foods by 4 months.

Model	Healthy Start			NorthPop		
	*N*	Hazard ratio (95% CI)	*p* value	*N*	Hazard ratio (95% CI)	*p* value
Unadjusted	1253	0.36 (0.27, 0.49)	< 0.0001	6446	0.70 (0.50–0.98)	0.037
Partially adjusted	1252	0.38 (0.28, 0.51)	< 0.0001	6155	0.72 (0.51–1.01)	0.055
Fully adjusted	945	0.41 (0.29, 0.57)	< 0.0001	4655	0.84 (0.55–1.29)	0.428

## 4 Discussion

In this study, using population‐based prospective data from the Healthy Start and NorthPop birth cohorts, a higher maternal diet index (MDI) score in pregnancy was associated with a lower hazard of physician‐diagnosed asthma in offspring in both cohorts, but only in unadjusted models. After adjustment for maternal asthma, a strong risk factor for offspring asthma [28], the HR was attenuated in NorthPop and no longer reached statistical significance, and in the fully adjusted model, MDI was not significantly associated with offspring asthma. In the Healthy Start population, a higher MDI, dichotomized by the median, was significantly associated with lower hazard of offspring asthma also in the fully adjusted model. The persistence of significance in Healthy Start but not in NorthPop may be explained by difference in diet variability, childhood asthma incidence, or confounder distributions. Hence, potential effect modification by population context or dietary pattern composition may explain the loss of significance after multivariable adjustments in NorthPop.

The lack of association in NorthPop suggests that the MDI requires further external validation and investigations in other populations where covariates collected are more similar. It also raises questions about whether diet indices that are population‐specific should be developed.

The observation of a protective maternal dietary pattern in pregnancy in Healthy Start echoes some earlier work on overall diet quality and childhood respiratory outcomes. Adherence to Mediterranean‐style diets in pregnancy has been linked to lower wheeze and atopy in offspring [29], while higher diet diversity has shown inverse associations with asthma [15]. Intervention with a low glycemic index (GI) diet in pregnancy have yielded inverse association to childhood asthma in one study [30]. Further, a recent systematic review concluded that a pro‐inflammatory diet in pregnancy is linked to increased asthma/wheeze risk in children under 5 years of age but with high heterogeneity across studies [31]. However, positive associations and null findings for childhood respiratory disease and asthma have also been repeatedly demonstrated for well documented dietary patterns like the Mediterranean diet score [6, 7], the Alternative Healthy Eating Index [7], and the Dietary Inflammatory Index [8, 9, 10, 32]. Importantly, these widely used dietary indexes were originally developed based on overall adult populations and mainly focused on their relation to non‐communicable diseases and survival in middle aged or an elderly population [11, 12, 13]. Hence, they were not promoted based on maternal diet in pregnancy or to offspring immune development.

The inclusion of specific foods in MDI—yogurt and vegetables inversely associated with childhood asthma, and fries, juice, red meat, cold cereals, and rice positively associated with child asthma appears to capture key components for asthma prevention based on earlier studies [14, 15, 16, 17, 18], which these broader patterns may not capture [6, 7, 8, 9, 10, 32]. Although we could not find an association between MDI and decreased offspring asthma incidence in NorthPop after adjustments for potential confounders, similar associations between components of MDI and asthma outcomes have been found in previous studies. For example, Baϊz et al. showed positive associations between meat intake and wheeze [33], Wright et al. showed positive associations between sugar‐sweetened beverages/total fructose and current asthma [34], and Ogawa et al. showed inverse associations between cruciferous (but not total) vegetables and asthma [35].

Dietary prevention remains an intriguing and biologically plausible approach to reduce offspring asthma risk. Early life exposure to a highly diverse microbiome is suggested to protectively influence on asthma risk [2] and optimizing maternal nutrition in pregnancy has good potential for prevention [36, 37]. It has been demonstrated that diet plays a central role in determining the composition of gut microbiota in pregnancy [38] and in turn, the maternal gut microbiome may modulate offspring immune development [39, 40]. Although this observational study cannot establish causality, several potential mechanisms may underline the unadjusted association between maternal diet and offspring asthma in both study populations. Maternal diet‐induced alterations in the gut microbiota can interact with the fetoplacental unit, facilitating the transfer of microbial antigens and metabolites, such as short‐chain fatty acids (SCFAs), across the placenta to influence fetal immune development [41]. SCFAs in particular are known to promote regulatory T‐cell differentiation and anti‐inflammatory cytokine profiles in the neonate [42]. Notably, along with dietary fiber, resistant starch and oligosaccharides found in a diverse diet and equivalent to a high MDI score, including higher intake of for example wholegrains, pulses, kale, onion and other vegetables, provide substrates for microbiota‐accessible carbohydrates [43]. Additionally, immunoglobulin‐mediated transport of bacterial components and cytokine signaling may align maternal and fetal immunity toward a less pro‐allergic phenotype [41].

Both the Healthy Start and Northpop studies have previously shown that a more diverse diet in pregnancy is inversely associated with risk of offspring allergy outcomes (i.e., allergic rhinitis, atopic dermatitis, wheeze, food allergy) and asthma [15, 21]. Supporting the importance of a healthy diverse diet in asthma prevention, three studies indicate that an increased inflammatory diet pattern in pregnancy is associated with childhood asthma and/or wheeze in children under 5 years [9, 32, 44]. These studies may indicate a potential biological interaction between diet diversity or a healthier diet and the Dietary Inflammatory Index: a diverse diet may reduce the inflammatory potential of the diet, while a monotonous, nutrient‐poor diet may amplify inflammatory signaling.

Strengths of this study include the high number of included mother‐child dyads, the high number of asthma cases seen in both cohorts, the long follow‐up and prospective design of both cohorts included, the alignment between the recording of maternal diet, the robust registry‐based asthma ascertainment via ICD‐10 coding in the large NorthPop study population [26] and the use of medical record searching in Healthy Start. Further, the consistency of results using the ≥ 2 asthma‐coded contacts definition in NorthPop provides reassurance regarding the robustness of our findings despite differences in outcome ascertainment between the two cohorts. The use of adjusted parametric Weibull time‐to‐event models could account for censoring and assess both incidence and timing in this study.

Dietary intake was self‐reported in both cohorts which indicate a limitation. In Healthy Start, women were asked to complete FPQs at their mid‐pregnancy and delivery research visit—then data from the two FPQs were combined by computing the maximum of the two responses. In NorthPop, one FFQ was completed around gestational week 35. The association may be attenuated due to this discrepancy.

As the Healthy Start population was followed until at least 4 years of age and NorthPop is still longitudinally collecting data from the included families, the median follow‐up was shorter for the NorthPop sub‐population used in this study (i.e., median 0.9 years shorter in the fully adjusted model). Future studies will enable more detailed assessment of age‐specific associations, for example, including comparisons between preschool and school‐age asthma. Asthma in NorthPop was identified through ICD‐10 J45x codes but without asthma medication data because of a delay in prescription data delivery.

We dichotomized MDI using the Healthy Start median to facilitate direct comparison to previous and future studies. The imbalance in NorthPop (only 6.1% below Healthy Start median) illustrates the great differences in dietary intake across US and Swedish populations and the potential need to either use cohort‐specific cut points or continuous measurement. However, a dose‐response relationship for dietary patterns in relation to health outcomes is rarely expected while comparing two distinct groups may be more generalizable [45]. Freedman et al. provide strong evidence that dietary measurement error often masks true dose‐response effects [46], supporting the rationale for using dichotomizing of the MDI.

Residual confounding by unmeasured factors (e.g., later childhood environmental exposures) cannot be excluded. Given the high proportion of women with at least some university education, generalizability may be limited to similar high‐educated Northern European and US populations. Based on the differences in population characteristics and MDI distribution seen between the two cohorts, NorthPop may not be the most appropriate population for external validation of the MDI.

Without further external validation of the MDI, modifications of present dietary recommendations for pregnant women cannot be made. Future observational studies in other populations and settings together with mechanistic sub studies of maternal and/or infant microbiota and SCFA profiling could provide more evidence. Such efforts would advance primary prevention strategies for childhood asthma, which remains a leading lifelong chronic disease in both the United States [1] and Sweden [2].

## Author Contributions

**Stina Bodén:** conceptualization, data curation, formal analysis, funding acquisition, methodology, project administration, visualization, writing – original draft, writing – review and editing. **Carina Venter:** conceptualization, data curation, investigation, methodology, writing – review and editing. **Kaci Pickett‐Nairne:** conceptualization, formal analysis, methodology, validation, writing – review and editing. **Deborah H. Glueck:** conceptualization, formal analysis, methodology, validation, writing – review and editing. **Richard Lundberg‐Ulfsdotter:** data curation, resources, writing – review and editing. **Magnus Domellöf:** resources, investigation, funding acquisition, project administration, writing – review and editing. **Dana Dabelea:** funding acquisition, resources, methodology, project administration, writing – review and editing. **Christina E. West:** funding acquisition, resources, methodology, project administration, supervision, validation, writing – review and editing.

## Funding

This study was funded by the Swedish Research Council Grants 2018‐02642 and 2021‐01367 (C.E.W.); the Heart‐Lung Foundation Grant 20180641 (C.E.W.); the Ekhaga Foundation Grant 2018–40 (C.E.W.); the Västerbotten County Council (A.L.F.) Grants RV 832441, RV 840681 (C.E.W.), and RV‐960756 (S.B.), Umeå University’s strategic research funds (S.B.), and the Unit of Research, Region Jämtland Härjedalen Foundation Grant JLL‐993235 (S.B.) and Grant JLL‐993810 (S.B.). S.B has received scholarships from the Swedish Foundation for International Cooperation in Research and Higher Education (STINT) and from Henning och Johan Throne‐Holst's foundation for the promotion of scientific research. The NorthPop infrastructure receives funding from Västerbotten County Council, Umeå University, The Kempe Foundation and the Forte government agency (C.E.W. and M.D.). This work was also supported by the National Institutes of Health, Grants R01 DK076648/DK/NIDDK NIH HHS/United States, R01 GM121081/GM/NIGMS NIH HHS/United States, UG3 OD023248/OD/NIH HHS/United States, UH3 OD023248/OD/NIH HHS/United States, R25GM111901‐S1, R25GM111901. The funding bodies had no role in study design, data collection and analysis nor in the preparation of the manuscript.

## Conflicts of Interest

Dr. Venter reports grants from Reckitt Benckiser Food Allergy Research and Education, National Peanut Board; personal fees from Reckitt Benckiser, Nestle Nutrition Institute, Danone, Abbott Nutrition, Else Nutrition, Before Brands and Owen outside the submitted work. Dr. West has received research funding from Thermo Fisher Scientific and Arla, which was directly paid to the institution, and speaker honoraria from Thermo Fisher Scientific, Aimmune Therapeutics, a Nestlé Health Science company, and Nutricia outside the submitted work. The other authors declare no conflicts of interest. Dr. Domellöf has received consultation honoraria or speaker fees Baxter Medical AB, Danone Nutricia, Elgan Pharma, Fresenius Kabi Deutschland GmbH, Intertek Health Sciences Inc. and Ipsen AB as well as a grant/research support from Arla Foods Ingredients.

## Supporting information


Supporting Information S1

## Data Availability

Data available on request due to privacy/ethical restrictions.
