# Supplementary material for: Associations Between the Maternal Diet Index and Childhood Asthma: The NorthPop and Healthy Start Cohorts
Source: Clin Transl Allergy. 2025 Dec 18;15(12):e70144. doi: 10.1002/clt2.70144 (PMC12713374; doi:10.1002/clt2.70144)
Supplement: Supplementary file 1 — Supporting Information S1 [file CLT2-15-e70144-s001.pdf]

Supplemental material

Associations between the maternal diet index and childhood asthma: the NorthPop and Healthy Start cohorts

**Supplemental tables:**	**Description**	**Page**
Supplemental Table S1	Characteristics of Healthy Start study population	2
Supplemental Table S2	Characteristics of NorthPop study population	3
Supplemental Table S3	Descriptives of food frequency intake in the Healthy Start study population	4
Supplemental Table S4	Descriptives of food frequency intake in the NorthPop study population	5
Supplemental Table S5	Healthy Start childhood asthma incidence data	6
Supplemental Table S6	NorthPop childhood asthma incidence data	7
Supplementary Table S7	NorthPop association between MDI and childhood asthma determined with ≥2 J45x ICD-10 codes	8
Supplementary Table S8	NorthPop associon between MDI dichotomized by the cohort-specific median (74.1) and childhood asthma determined with ≥1 J45x ICD-10 code	8

Supplemental Table 1. Characteristics of the Healthy Start study population of pregnant women and dichotomized by the median of Maternal diet index (MDI) in pregnancy (72.2).

Characteristics	n (%)/mean (SD)	missing, n (%)	n (%)/mean (SD)		p-value [a]
	All		MDI <72.2	MDI >72.2	
**Mother-child dyads, n**	**1253**		**627**	**626**	
**Categorical variables, n (%)**					
Maternal university education [b]	553 (44.1)	0 (0.0)	138 (22.0)	415 (66.3)	<0.0001
Maternal history of asthma	208 (16.6)	1 (0.1)	122 (19.5)	86 (13.7)	0.008
Maternal smoking in pregnancy	106 (8.5)	0 (0.0)	82 (13.1)	24 (3.8)	<0.0001
First born child	594 (47.4)	0 (0.0)	311 (49.6)	283 (45.2)	0.13
Caesarean section	270 (21.8)	14 (1.1)	143 (23.1)	127 (20.5)	0.25
Child sex girl	600 (47.9)	0 (0.0)	305 (48.6)	285 (47.1)	0.61
Any breastfeeding at age 6 months	672 (61.4)	159 (12.7)	238 (47.0)	433 (74.0)	<0.0001
Early (<4 months) introduction of solid food	149 (15.1)	265 (21.1)	80 (18.0)	69 (12.7)	0.03
**Continuous variables, mean (SD)**					
Maternal BMI (kg/m2)	25.7 (6.2)	0 (0.0)	26.5 (6.6)	25.1 (5.7)	<0.0001
Maternal age at delivery (years)	27.8 (6.2)	0 (0.0)	25.1 (5.9)	30.5 (5.3)	<0.0001
Gestational age at birth (weeks)	39.3 (1.7)	0 (0.0)	39.1 (1.8)	39.4 (1.6)	<0.0001

[a] P-value from Fischer's exact test for categorical variables and ANOVA for continuous variables for comparison between groups of MDI, dichotomized by the median from the source population (Healthy Start study) which was 72.2.
[b] Any university level education

BMI, body mass index; MDI, maternal diet index

Supplementary Table 2. Characteristics of the NorthPop Birth Cohort study population of pregnant women and dichotomized by the median of Maternal diet index (MDI) in pregnancy (72.2) from the source population Healthy Start.

**Characteristics**	**n (%)/mean (SD)**	**missing, n (%)**	**n (%)/mean (SD)**		**p-value** [a]
	**All participants**		**MDI <72.2**	**MDI >72.2**	
**Mother-child dyads, n**	**6446**		**391**	**6055**	
**Categorical variables, n (%)**					
Maternal university education [b]	4092 (63.5)	276 (4.3)	162 (41.4)	3930 (64.9)	<0.001
Maternal history of asthma	1132 (17.6)	291 (4.5)	88 (23.7)	1044 (18.1)	0.009
Maternal smoking in pregnancy	75 (1.2)	234 (3.6)	10 (2.6)	65 (1.1)	0.023
First born child	3017 (46.8)	25 (0.4)	188 (48.1)	2829 (46.7)	0.600
Caesarean section	1062 (16.5)	25 (0.4)	66 (16.9)	996 (16.4)	0.833
Child sex girl	3172 (49.2)	0 (0.0)	188 (48.1)	2984 (49.3)	0.676
Any breastfeeding at age 6 months	4319 (82.7)	1223 (19.0)	224 (73.7)	4095 (83.2)	<0.001
Early (<4 months) introduction of solid food	490 (7.6)	1506 (23.4)	45 (11.5)	445 (7.3)	0.002
**Continuous variables, mean (SD)**					
Maternal BMI (kg/m2)	25.2 (4.8)	142 (2.2)	26.0 (5.7)	25.1 (4.8)	<0.001
Maternal age at delivery (years)	31.0 (4.4)	0 (0.0)	29.4 (4.7)	31.1 (4.3)	<0.001
Gestational age at birth (weeks)	39.5 (1.4)	0 (0.0)	39.3 (1.4)	39.5 (1.4)	0.009

[a] P-value from Fischer's exact test for categorical variables and ANOVA for continuous variables for comparison between groups of MDI, dichotomized by the median from the source population (Healthy Start study) which was 72.2.
[b] Any university level education

BMI, body mass index; MDI, maternal diet index

Supplementary Table 3. Descriptives of food frequency intake in the Healthy Start study population of pregnant women of seven foods included in the MDI and macronutrient intake, dichotomized by the MDI median.

	**HEALTHY START POPULATION**		
	**MDI <72.2 (n=627)**	**MDI >72.2 (n=626)**	**p-value**
**Foods included in MDI**			
Fries	0.42 (0.39)	0.13 (0.1)	<0.0001
Yogurt	0.38 (0.42)	0.68 (0.45)	<0.0001
Juice	1.38 (1.48)	0.51 (0.6)	<0.0001
Cold cereal	0.89 (0.59)	0.49 (0.34)	<0.0001
Red meat	0.52 (0.45)	0.27 (0.23)	<0.0001
Rice	0.48 (0.44)	0.33 (0.29)	<0.0001
Vegetables	0.66 (0.52)	1.16 (0.68)	<0.0001
**Macronutrients**			
Energy intake, kcal/day	2100.16 (431.36)	2024.09 (326.52)	0.0005
Carbohydrates, gram/day	263.76 (110.28)	248.57 (74.12)	0.005
Fat, gram/day	83.23 (39.68)	77.47 (27.71)	0.003
Protein, gram/day	82.8 (33.87)	82.2 (25.43)	0.73
Fiber, gram/day	16.5 (8.34)	20.14 (8.23)	<0.0001

MDI, maternal diet index

Supplementary Table 4. Descriptives of food frequency intake in the NorthPop study population of pregnant women of seven foods included in the MDI and macronutrient intake, dichotomized by the MDI median from the source population Healthy Start.

	**NORTHPOP POPULATION**		
	**MDI <72.2 (n=391)**	**MDI >72.2 (n=6055)**	**p-value**
**Foods included in MDI**			
Cold cereal [a]	0.37 (0.71)	0.16 (0.34)	<0.001
Fries [b]	0.29 (0.36)	0.14 (0.16)	<0.001
Juice [c]	0.70 (0.99)	0.40 (0.57)	<0.001
Red meat [d]	1.86 (1.38)	0.74 (0.56)	<0.001
Rice [e]	0.46 (0.73)	0.35 (0.27)	<0.001
Vegetables [f]	1.89 (1.33)	3.39 (1.85)	<0.001
Yogurt [g]	0.34 (0.45)	0.84 (0.81)	<0.001
**Macronutrients**			
Energy intake, kcal/day	2535 (1165.99)	2172.38 (752.58)	<0.001
Carbohydrates, gram/day	282.72 (137.18)	262.64 (96.49)	<0.001
Fat, gram/day	92.72 (48.91)	73.27 (28.50)	<0.001
Protein, gram/day	129.66 (44.28)	99.57 (40.28)	<0.001
Fiber, gram/day	21.81 (14.51)	27.45 (14.72)	<0.001

The following foods were included in the seven food components in the NorthPop data:
[a] Corn white flakes, K-special, and sweet cereals like Frosties
[b] French fries, pommes frites, fried potatoes
[c] Fruit juice
[d] Beef, pork, lamb, game, and black pudding
[e] White rice, brown rice, cracked wheat (bulgur)
[f] Cucumber, avocado, bell pepper, tomatoes, cauliflower, broccoli, white cabbage, carrots and other root vegetables, corn, onion, mushrooms, and frozen vegetables
[g] Sweetened and unsweetened yogurt and Kefir (i.e. sour milk)

MDI, maternal diet index

Supplemental Table 5. Healthy Start childhood asthma incidence data per time-to-event model with number (n) of asthma cases, total (n) particpants, total person-year and median person-year for all particpants and if below or above the median of 72.2.

	**All**	**<MDI 72.2**	**>MDI 72.2**
**Crude unadjusted model**			
n childhood asthma	215	153	62
n all	1253	627	626
person-year, total	6194	2892	3301
person-year, median (IQR)	4.9 (4.0, 6.5)	4.0 (4.0, 6.2)	5.2 (4.0, 6.6)
**Partially adjusted model** [a]			
n childhood asthma	215	153	62
n all	1252	626	626
person-year, total	6189	2887	3301
person-year, median (IQR)	4.9 (4.0, 6.5)	4.0 (4.0, 6.3)	5.2 (4.0, 6.6)
**Multivariable adjusted model** [b]			
n childhood asthma	156	106	50
n all	945	417	528
person-year, total	4772	1955	2816
person-year, median (IQR)	5.2 (4.0, 6.4)	4.8 (4.0, 6.3)	5.4 (4.0, 6.5)

[a] Adjusted for maternal history of asthma

[b] Adjusted for maternal history of asthma, gestational smoking (yes/no), gestational energy intake, if first parity (yes/no), mode of delivery (ceasarian section or vaginal), child sex (boy/girl), any breastfeeding at age 6 months, and if early introduction of solid foods (<4 months of age)

IQR, inter quartile range; MDI, maternal diet index

Supplemental Table 6. NorthPop childhood asthma incidence data per time-to-event model with number (n) of asthma cases, total (n) particpants, total person-year and median person-year for all particpants and if below or above the median of the source population Healthy Start.

	**All**	**<MDI 72.2**	**>MDI 72.2**
**Crude unadjusted model**			
n childhood asthma	455	37	418
n all	6446	391	6055
person-year, total	27692	1639	26054
person-year, median (IQR)	4.2 (2.7, 5.8)	4.2 (2.8, 5.6)	4.2 (2.7, 5.9)
**Partially adjusted model** [a]			
n childhood asthma	439	36	403
n all	6155	372	5783
person-year, total	26 592	1566	25026
person-year, median (IQR)	4.3 (2.7, 5.9)	4.2 (2.8, 5.9)	4.3 (2.7, 5.9)
**Multivariable adjusted model** [b]			
n childhood asthma	334	23	311
n all	4655	279	4376
person-year, total	20329	1165	19164
person-year, median (IQR)	4.3 (2.7, 5.8)	4.2 (2.7, 5.5)	4.3 (2.8, 5.9)

[a] Adjusted for maternal history of asthma

[b] Adjusted for maternal history of asthma, gestational smoking (yes/no), gestational energy intake, if first born child (yes/no), mode of delivery (caesarean section or vaginal), child sex (boy/girl), any breastfeeding at age 6 months, and if early introduction of solid foods (<4 months of age)

IQR, inter quartile range; MDI, maternal diet index

**Supplementary Table 7.** Maternal diet index (MDI) in NorthPop dichotomized by the Healthy Start median (72.2) in pregnancy and unadjusted and adjusted associations with hazard of childhood asthma diagnosis determined with at least two J45x ICD-10 codes (n=319). The partially adjusted model adjusts for history of maternal asthma. The fully adjusted model additionally adjusts for gestational smoking, gestational energy intake, caesarean section, first born child, child sex, any breastfeeding at age 6 months, and introduction of solid foods by 4 months.			
	**NorthPop**		
**Model**	**N**	**Hazard ratio (95% CI)**	**p-value**
Unadjusted	6446	0.58 (0.40-0.83)	0.004
Partially Adjusted	6155	0.59 (0.41-0.86)	0.007
Fully Adjusted	4655	0.65 (0.41-1.02)	0.064

**Supplementary Table 8.** Maternal diet index (MDI) in NorthPop dichotomized by the cohort-specific median (74.71) in pregnancy and unadjusted and adjusted associations with hazard of childhood asthma diagnosis determined with at least one J45x ICD-code (n=455). The partially adjusted model adjusts for history of maternal asthma. The fully adjusted model additionally adjusts for gestational smoking, gestational energy intake, caesarean section, first born child, child sex, any breastfeeding at age 6 months, and introduction of solid foods by 4 months.			
	**NorthPop**		
**Model**	**N**	**Hazard ratio (95% CI)**	**p-value**
Unadjusted	6446	0.97 (0.81-1.17)	0.761
Partially Adjusted	6155	0.99 (0.82-1.19)	0.899
Fully Adjusted	4655	1.04 (0.83-1.29)	0.739
